# Intraoperative Kirschner Wire Breakage in a Pediatric Supracondylar Humerus Fracture

**DOI:** 10.7759/cureus.13794

**Published:** 2021-03-10

**Authors:** Gaurav Ardawatia, Ankit B Waghela, Ashish S Ranade

**Affiliations:** 1 Orthopaedics, Deenanath Mangeshkar Hospital and Research Centre, Pune, IND; 2 Blooming Buds Centre for Pediatric Orthopaedics, Deenanath Mangeshkar Hospital and Research Centre, Pune, IND

**Keywords:** complications, instrument breakage, k wire, supracondylar humerus fracture, pediatric, intraoperative

## Abstract

A displaced supracondylar humerus in a child is usually treated with closed reduction and percutaneous Kirschner (K)-wire fixation. The procedure is straightforward and usually yields excellent outcomes. In general, intraoperative complications are uncommon and intraoperative complications related to K-wires are exceedingly rare. We present the case of intraoperative K-wire breakage while performing closed reduction and K-wire fixation for a pediatric supracondylar humerus fracture. This unusual complication occurred while drilling through the medial cortex and the broken end of the K-wire disappeared under the skin in the cartilaginous distal humerus. The broken wire was removed by making an incision over the broken end. This report serves as a reminder to follow principles of drilling and avoid K-wire-related complications while performing percutaneous fixation of the pediatric supracondylar humerus fracture.

## Introduction

In children, supracondylar humerus fractures are common. Closed reduction with K-wire fixation is the most commonly practiced treatment modality for the displaced fractures [1]. The majority of the patients recover well without any complications or deficits. Wire backout, infections, and injury to the neurovascular structures are the common complications observed [2]. Intraoperative breakage of K-wire in the supracondylar humerus fracture in children has not been reported. We present a case where an intraoperative K-wire breakage and uncommon complication were encountered while performing closed reduction with K-wire fixation for a displaced supracondylar humerus fracture in a child.

## Case presentation

**Figure 1:**
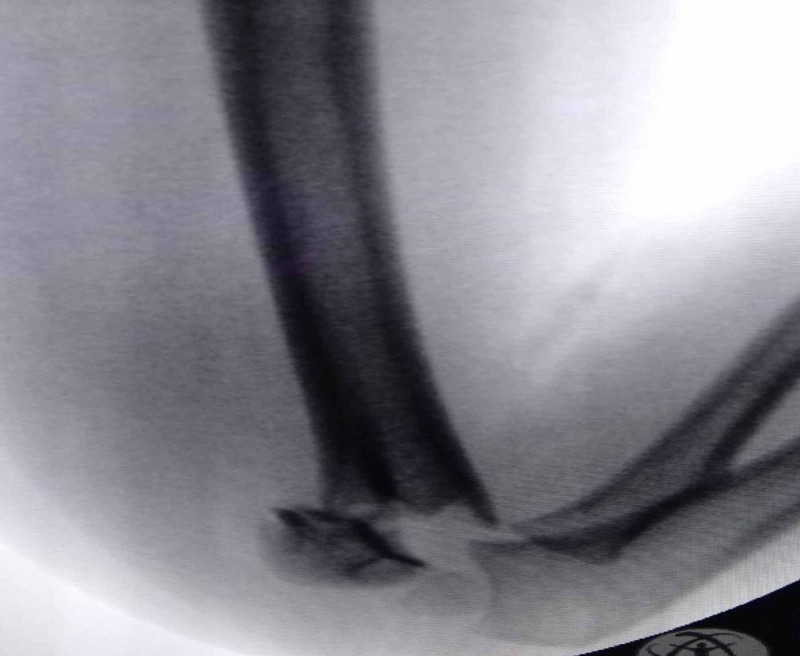
Preoperative Image 1

**Figure 2:**
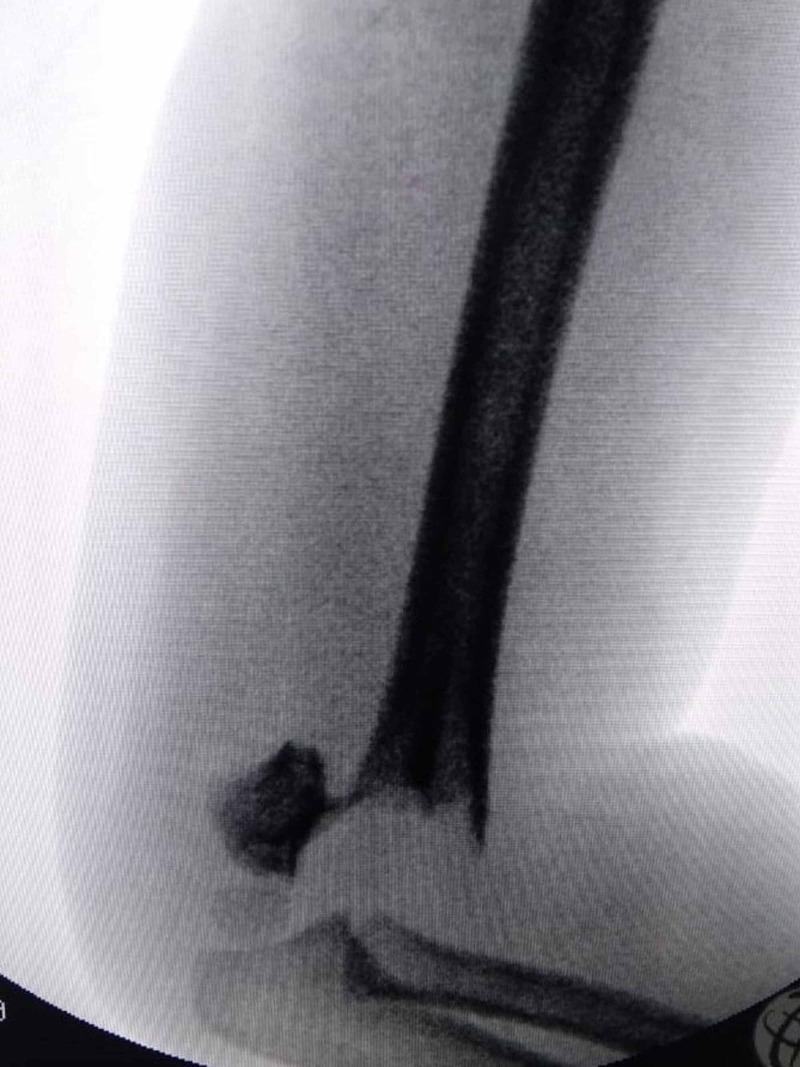
Preoperative Image 2

A four-year-old male was admitted to our hospital with left elbow swelling and pain. Plain radiography in anteroposterior and lateral view revealed a Gartland type 3 left supracondylar humerus fracture (Figure 1, 2).

**Figure 3:**
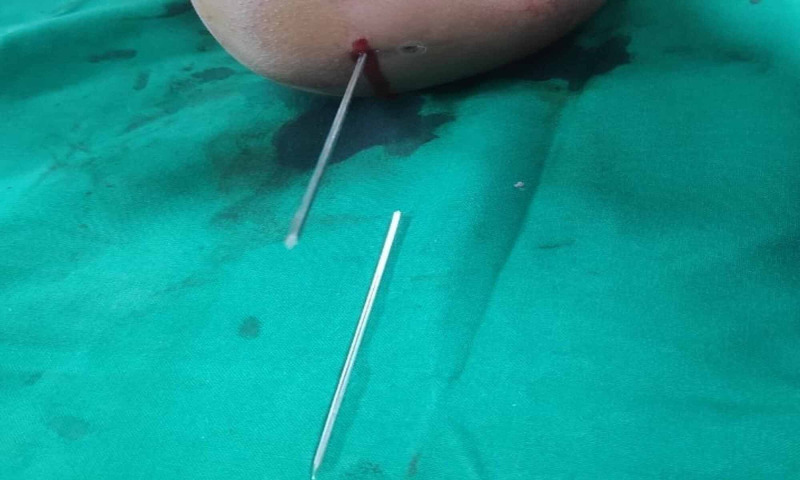
Intraoperative image showing one K-wire holding the fracture reduction and second broken K-wire lying on operative table

**Figure 4:**
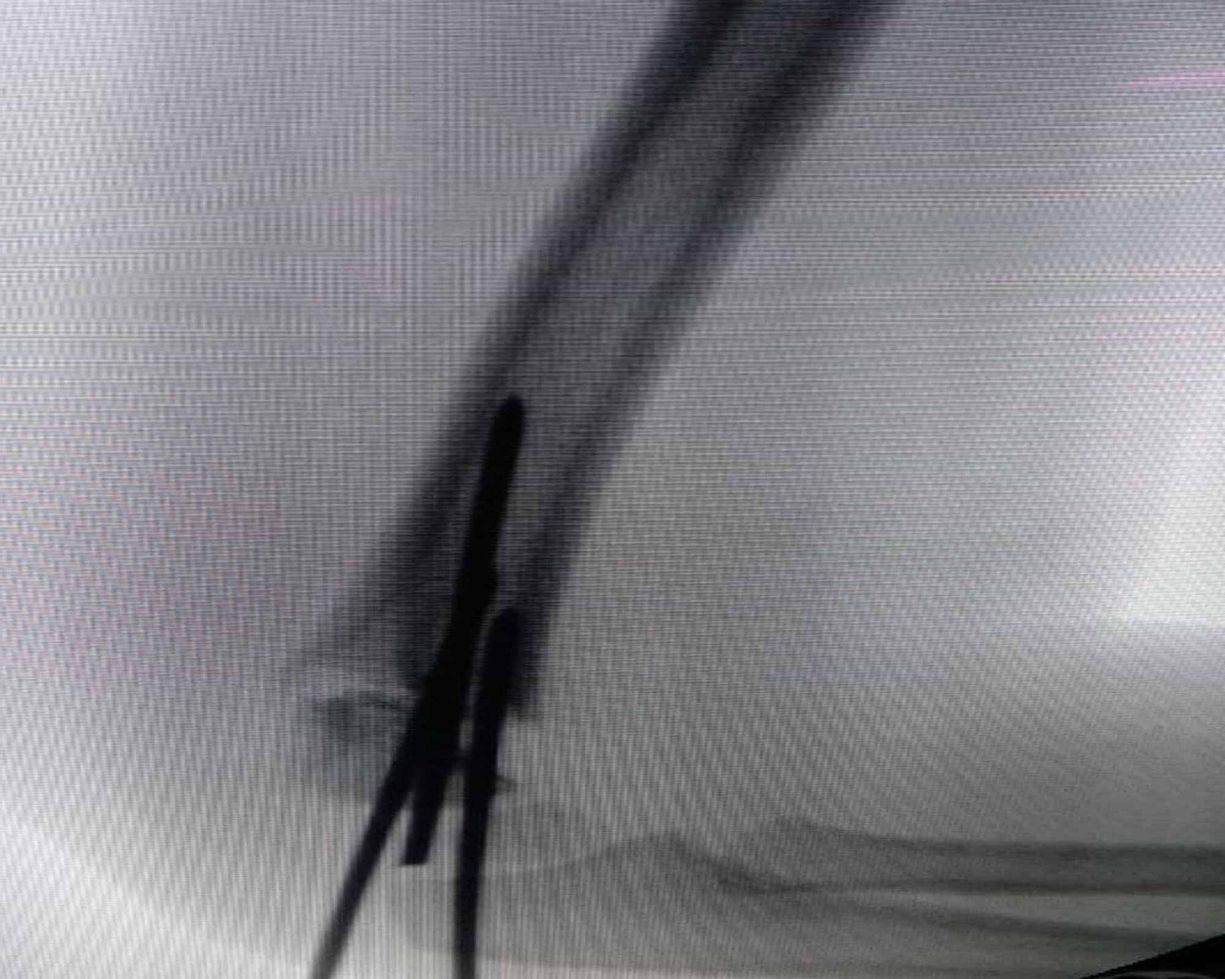
Intraoperative C arm image with two K-wires holding the fracture with one broken K-wire in middle.

There was no neurological deficit. Under general anesthesia, using c arm control, closed reduction of the fracture was done by an orthopedic trainee under consultant supervision. After maintaining the reduction in hyperflexion and pronation of the elbow, a 1.6 mm diameter K-wire was inserted using the lateral entry point. After drilling the lateral column, it was felt that the wire was skiving proximally in the medullary canal, hence, further drilling was done to obtain purchase in the medial cortex. While the wire was being drilled it broke near the entry point and the broken end disappeared under the skin, leaving the remnant portion buried in the distal humerus (Figure 3). Since the reduction was acceptable in anteroposterior and lateral views, three more 1.6mm K-wires were inserted from the lateral to the medial side (Figure 4).

**Figure 5:**
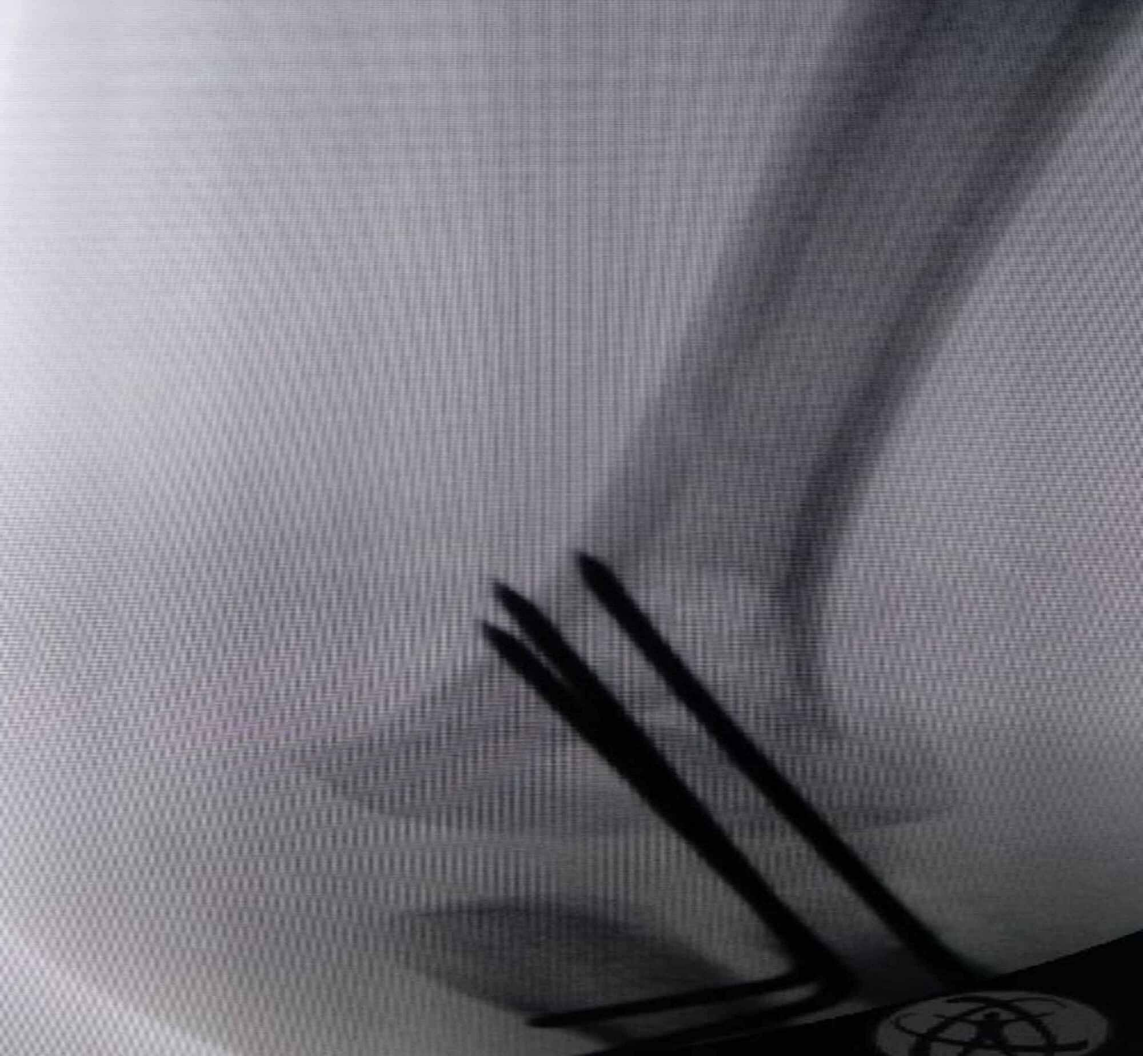
Final intraoperative c-arm image after fixation of fracture with three K-wires and removal of broken K-wire.

Fixation of fracture was confirmed under fluoroscopy. After fracture fixation, attempts were made to remove the broken K-wire but it was not possible to grab the tip, as the broken end was buried under the cartilaginous bone. At this point, intraoperatively we scrubbed out and discussed complications with the family and after taking their consent we proceeded further under the same anesthesia. A skin incision was made over the broken K-wire, surrounding tissue and bone were cleared off and the tip was exposed. Using a needle nose plier, the tip was held and the wire removed in a rotatory motion and confirmed under c-arm (Figure 5). An above elbow slab was given for further stability. At one week postoperatively, the patient was found comfortable in the slab without any complaints. Follow-up at four weeks showed radiologic union and removal of K-wires was done in the clinic. The patient had a full range of motion of the elbow at three months. Further follow-ups were uneventful.

## Discussion

We encountered an unusual complication during a routine method of fracture fixation. Several case reports have described instrument breakage but intraoperative breakage of K-wire in the supracondylar humerus fracture in a pediatric patient has not been reported before.

The orthopedic community is widely using K-wires in various operations due to their simplicity, reliability, and cost-effectiveness. However, certain complications like loss of reduction, K-wire migration, damage to surrounding neurovascular structures, pin tract infection, and skin tethering are likely to happen. K-wire breakage is a rather rare event but can happen especially when the wire touches another metal or K-wire while crossing each other, not allowing them to progress inside, or in the postoperative period when we transfix the joint to prevent movements without supplemental fixation with a cast or splint [3].

There are studies in the literature describing the breakage of K-wire intraoperatively in the adult population but none in children. A study conducted by Price et al. reported 12 out of the 14 breakages occurred during trauma operations with an average age of 47 years and an overall instrument breakage rate of 0.18% [4].

Pichler et al. reported three out of 42 cases of instrument breakage occurred during elective surgery. The study also did not observe a high rate of instrument breakage by any specific orthopedic implant [5].

Hong et al. reported breakage of K-wire in a young male during removal. The complication was pin-related rather than fracture fixation-related, which arose due to defective manufacturing of K-wire [6]. Similarly, Wong et al. reported breakage of a K-wire in the middle phalanx while removing it from a metacarpal shaft fracture in an adult male. A broken K-wire was removed by extended dissection of the extensor tendon on the ulnar side of the middle phalanx to avoid chances of osteomyelitis at a later stage [7].

Intraoperatively broken K-wires were removed at a later stage in most of the studies. Roy et al. reported breakage and retrieval of the guide pin in midfoot surgery in a 20-year-old woman, which was removed after two weeks of index surgery [8]. These may not be suitable for pediatric patients considering the growing skeleton, as the smooth broken wire needs removal as it has a risk of migration, articular surface damage, physeal arrest, and damage to the surrounding neurovascular structures. Removal of remaining wire fragments indeed poses a great challenge because the site of breakage is deep within the bone, as obtaining accurate purchase of the broken fragment without damage to adjacent bones and joint tissues is difficult. It would be prudent to avoid intraoperative K-wire breakage. The literature describes some of the ways to avoid K-wire-related complications.

As per Aernout and co-authors, positioning the index finger of the opposite hand just behind the aimed exit point when drilling in the bone improves the results due to the benefit of proprioception [9]. Also during drilling, if the angle is kept more than 45° measuring from the horizontal articular surface (also called the minimal critical angle), it avoids slippage of the wires over the opposite cortex. The study observed that wires perforate easily and pass opposite cortices when inserted at ≥45° [10]. Lobst et al. have reported that when a smaller diameter wire was used at lower inclinations, the angle of contact with the far cortex may be large causing the wire not to penetrate the cortex and leading to slippage of the wire tip over the endosteal surface of the opposite cortex. A smaller diameter wire might give flexibility during the reduction of fracture fragment, it has a higher chance of breakage, and also it allows it to bounce off the far cortex and travel upwards in the medullary canal without achieving bicortical purchase [11]. Mechanically, repeated kinking and bending of the K-wire while drilling causes loss of strength, making it weaker and more likely to fracture.

In our case, the wire breakage happened because it was inserted at a high angle. It probably was engaged in cortical bone even though it appeared to be intramedullary on the c-arm, while we were aiming to change the direction by withdrawing it and drilling it at a lower angle. During this maneuver, the direction of push changed after the pin was already in the far cortex, causing it to bend at the near cortex and then fatigue and break, as the tip of the wire at the far end was engaged in the cortex.

## Conclusions

K-wire breakage is an important complication of orthopedic surgery, and if such complications are avoidable during surgical procedures, it is important to recognize the ways to minimize and prevent this complication.

Some of the techniques to avoid breakage of K-wires are: using a greater diameter K-wire of 2.0mm; not putting pressure on the K-wire, just keep drilling over the same area to avoid skiving; putting the K-wire at an inclination angle of 45° or more; aiming at the index finger of the opposite hand kept at the intended exit point while drilling; and avoiding previously used K-wire/over-drilled K-wires from the same or previous surgeries.
